# The impact of financial incentives on physical activity in adults: a systematic review protocol

**DOI:** 10.1186/s13643-018-0687-8

**Published:** 2018-01-25

**Authors:** My-Linh Nguyen Luong, Kim L. Bennell, Michelle Hall, Anthony Harris, Rana S. Hinman

**Affiliations:** 1 Centre for Health, Exercise and Sports Medicine, Department of Physiotherapy, The University of Melbourne, Level 7, Alan Gilbert Building, Building 104, 161 Barry St, Melbourne, VIC 3010 Australia; 2 Centre for Health Economics, Monash Business School, Monash University, 15 Innovation Walk, Level 2, Clayton, VIC 3800 Australia

**Keywords:** Systematic review, Financial incentive, Physical activity, Behaviour change, Public health, Review, Behavioural economics

## Abstract

**Background:**

Most adults fail to meet global physical activity guidelines set out by the World Health Organization. In recent years, behavioural economic principles have been used to design novel interventions that increase physical activity. Immediate financial rewards, for instance, can motivate an individual to change physical activity behaviour by lowering the opportunity costs of exercise. This systematic review will summarise the evidence about the effectiveness of financial incentive interventions for improving physical activity in adults.

**Methods:**

We will search MEDLINE, Embase, Cochrane Central Register of Controlled Trials, the Cumulative Index to Nursing and Allied Health Literature, Web of Science, Scopus, PsycINFO, EconLit, SPORTDiscus, the National Health Service Economic Evaluation Database, ClinicalTrials.gov and the World Health Organization International Clinical Trials Registry Platform from inception using a comprehensive, electronic search strategy. The search strategy will include terms related to ‘financial incentive’ and ‘physical activity’. Only randomised controlled trials that investigate the effect of financial incentives on physical activity in adult populations and that are written in the English language will be included. Two review authors will independently screen abstracts and titles, complete full text reviews and extract data on objective and self-reported physical activity outcomes. The authors will also assess the study quality using the Cochrane risk of bias tool and provide a systematic presentation and synthesis of the included studies’ characteristics and results. If more than two studies are sufficiently similar in population, settings and interventions, we will pool the data to conduct a meta-analysis. If we are unable to perform a meta-analysis, we will conduct a narrative synthesis of the results and produce forest plots for individual studies. Our subgroup analyses will examine the differential effects of an intervention in healthy populations compared to populations with disease pathology and compare the effects of interventions using financial rewards to interventions using financial penalties.

**Discussion:**

This systematic review will determine the effectiveness of positive and negative financial incentives on physical activity in adults. Findings will help inform the development of public health interventions and research in this field.

**Systematic review registration:**

PROSPERO 2017:CRD42017068263

**Electronic supplementary material:**

The online version of this article (10.1186/s13643-018-0687-8) contains supplementary material, which is available to authorized users.

## Background

Physical inactivity is one of the five leading global risks for mortality and a key factor that elevates the risk of chronic disease across all income groups [1]. One in three adults in high-income countries fails to meet the World Health Organization (WHO) physical activity recommendation to engage in 150 min of moderate aerobic exercise or 75 min of vigorous aerobic exercise, per week [2]. Since engaging in physical activity can reduce the burden of cardiovascular disease and type II diabetes and improve musculoskeletal symptoms and depression [1, 3], public health interventions aim to help all adults meet WHO physical activity recommendations.

Many factors influence a person’s participation in physical activity. Researchers use social ecological models to classify these factors into different levels of influence, which include intrapersonal factors, interpersonal processes, institutional factors, community factors and public policies [4]. Research suggests that interventions that target multiple levels of the social ecological model are most effective at changing behaviour [5]. Individual-level variables, such as age, sex, health status, self-efficacy and motivation, are consistently associated with physical activity [6]. Physical activity interventions consistently consider the role of these individual-level factors and interpersonal processes, and it is becoming more common to use a model that also considers how contextual factors affect physical activity [7–9].

The COM-B system [10] is a contemporary behaviour change model that can integrate factors at multiple levels. It hypothesises that behaviour change results from the interaction between *capability* (the individual’s psychological and physical capacity to engage in the activity concerned), *opportunity* (factors outside the individual that make the behaviour possible or prompt it) and *motivation* (brain processes that direct behaviour, including both *reflective* processes and *automatic* processes). Recently, public health and prevention policies have used behavioural economic principles that influence opportunity and motivation to increase participation in physical activity [11].

Behavioural economics considers traditional economic principles in combination with psychological, cognitive, emotional and social demands to help explain people’s inconsistent decision-making [12]. Traditional consumer theory assumes that people make rational decisions when presented with new information, whereas behavioural economics recognises that people often use heuristics, or mental shortcuts, to intuitively form judgements and make decisions [12]. These systematic deviations from rational decision-making result in cognitive biases, such as *present bias* and *loss aversion*. In the case of physical activity, individuals exhibit a *present bias* when they overemphasise the immediate financial and opportunity costs of exercise participation (e.g. physical discomfort, giving up leisure time) at the expense of the potential long-term health benefits that can be gained (e.g. improved health symptoms, weight loss) [13]. One way to balance present bias is to provide an immediate reward in the form of a financial incentive, which may take the form of cash, point-based systems or vouchers that can be exchanged for material goods. Although an immediate reward may lead to a change in behaviour, the prospect of losing something can be more motivating than gaining a reward; this phenomenon is referred to as *loss aversion* [14]. For example, in one study, participants were more likely to meet their daily step goals if they were penalised $1.40 (from a pre-allocated monthly incentive) for failing to achieve their daily goal than when they were rewarded an equivalent economic value for achieving that same goal [15]. These two cognitive biases highlight how inconsistent or irrational decision-making can be used to motivate health behaviour change through financial incentive structures.

Four systematic reviews and one narrative review have evaluated the effect of financial incentives on exercise, fitness or physical activity behaviours [16–20]; however, the confidence in the findings of these reviews is diminished based on critical appraisal using AMSTAR 2 [21]. None of the reviews registered a protocol prior to commencement of the review nor assessed the impact of publication bias. Of the five existing reviews, the systematic review by Mitchell and colleagues based on AMSTAR 2 criteria [16] is the most rigorous. Mitchell et al. conducted a systematic review and meta-analysis on exercise session attendance and concluded that positive financial incentives were associated with an increase in exercise session attendance for short-term interventions (i.e. 4–26 weeks) [16]. Despite the strengths of this review, it is limited in its generalisability. It has a limited focus on the single, specific behaviour of exercise attendance and explicitly excludes studies evaluating subsidies and disincentives. Significantly, the popularity of financial incentive interventions has risen considerably since the search conducted in 2012. The inclusion of new studies may alter the review conclusion regarding the effectiveness of these types of interventions.

Although newer systematic reviews exist, they have methodological flaws that limit confidence in their findings. Barte and Wendel-Vos investigated physical activity more broadly [18] and found that incentives offered as rewards for reaching physical activity goals had positive short-term effects on physical activity with diminished effects in the long-term [18], but incentives that lowered the costs of physical activity behaviour (e.g. gym membership) had no effect. However, the authors used a limited search strategy that did not include discipline-specific databases and it is likely that relevant studies may have been omitted. Although Strohacker et al. [17] searched a wider variety of databases, the screening process used by the authors was not completed in duplicate nor did they conduct a risk of bias assessment. Molema et al. limited their review to studies conducted only in healthcare settings [19] and also did not conduct a comprehensive literature search or a risk of bias assessment. Martin et al. produced the only review examining financial incentives to promote active travel; however, the authors included non-randomised studies and did not perform a systematic review [20]. The evidence from these reviews suggests that financial incentives at the individual level of the social ecological model appear to have some positive effects on physical activity levels and/or exercise adherence in the short-term, but this effect appears to vary by physical activity outcome, setting, length of follow-up time and incentive design. The methodological limitations of published reviews provide only limited confidence in their findings. Thus, there is a need for an up-to-date high quality systematic review on the effect of financial incentives on physical activity that provides an accurate and comprehensive summary of the best available evidence [16–19, 21].

There is increasing support for the use of financial incentives to motivate people to increase their participation in physical activity at multiple levels [22, 23]. Public policies like road pricing congestion charges have been linked to increased active travel [24, 25], intrapersonal factors and interpersonal processes were considered in the development of an intervention that used individual and team-based financial incentives to aid people in achieving a step goal [26], and a survey of large employers in the USA found that approximately 70% of employee wellness programs used financial incentives to increase physical activity participation or workplace performance [27]. The wide diversity of incentives used and behavioural outcomes measured and the unique characteristics of the settings, populations and individuals for whom the intervention is designed make it difficult to understand why some financial incentive interventions are effective at changing physical activity behaviour while others are not. If researchers provide an explicit theoretical model and clearly describe behaviour change techniques used in their intervention, then other researchers can better understand the mechanisms that make these interventions effective [28–30].

Many behaviour change interventions fail to provide a theory of change or adequately describe the mechanisms that explain why the intervention should lead to behaviour change [28, 31]. This lack of transparency is common in interventions and policies inspired by behavioural economics [32] and is a concern echoed in a systematic review that explored the use of financial incentives for physical activity in healthcare settings [19]. Molema et al. aptly noted that study authors frequently omitted a rationale that specified their motivations for selecting a financial incentive for this particular population [19]. Researchers can use the TIDieR checklist to ensure that they fully describe the intervention’s key features [33] and the Adams et al. framework to systematically characterise the design of the financial incentive [34]. Using these two tools will allow the research community to better understand the mechanisms that underpin financial incentive interventions, facilitate future intervention development and allow replicability of effective interventions [30, 35].

The primary objective of this study is to systematically review randomised controlled trials (RCTs) that evaluate the effect of financial incentive interventions on physical activity participation in adults. Secondary objectives are to describe (i) the structure and design attributes of different interventions using an established framework, (ii) the degree to which an intervention is theory-informed, (iii) behaviour change techniques used in financial incentive interventions and (iv) the types and number of ecological levels that these intervention programs target.

## Methods/design

We used the Methodological Expectations of Cochrane Intervention Reviews (MECIR) standards [36] and Preferred Reporting Items for Systematic Review and Meta-Analysis Protocols (PRISMA-P) 2015 checklist (http://www.prisma-statement.org/Extensions/Protocols.aspx) [37, 38] to guide the development of this protocol. The systematic review protocol was prospectively registered with the International Prospective Register of Systematic Reviews (PROSPERO) on 28 June 2017 (PROSPERO 2017:CRD42017068263). We will report any changes to our protocol in our published systematic review.

### Inclusion criteria

#### Types of studies

Only RCTs written in the English language will be considered for inclusion. Study selection criteria were established a priori using the Population, Intervention, Comparison, Outcome (PICO) framework [39]. Studies that fulfil the following eligibility criteria will be included:

- Population: Adults aged 18 years or older, with or without health conditions, disease and/or pathology.
- Intervention: Interventions that use a financial incentive to encourage physical activity. We define a financial incentive as any material reward or penalty applied to an individual or group with the purpose of encouraging participation in physical activity. Incentives may include, but are not limited to, cash rewards, point-based systems or vouchers that can be exchanged for material goods, tickets in lotteries for prizes, deposit contracts, taxes or subsidies. We will evaluate financial incentives in all settings, such as public health campaigns, workplace and occupational environments, insurance plans, government initiatives and community and healthcare settings. If feasible, we will group the interventions according to the direction of the financial incentive (i.e. positive gain or avoidance of penalty).
- Comparison: Any comparison group, provided the only difference between the comparison and intervention groups is the specific financial incentive strategy under investigation. No restriction will be placed upon the nature of the comparison group. Thus, eligible comparison groups may include a no intervention arm, usual care, a placebo/sham group or another active intervention group. Trials comparing two or more different financial incentives are eligible as long as all other treatment elements are similar across trial arms.
- Outcome: Studies reporting a parameter of physical activity that was measured both prior to randomisation and following intervention. We define physical activity according to the WHO definition of physical activity (i.e. ‘any bodily movement produced by skeletal muscles that requires energy expenditure’) [40]. This definition encompasses both incidental/general physical activity as well as participation in planned, structured, purposeful activity, such as a prescribed exercise program. Measures of physical activity at a population level are complex, and there is currently no consensus on a gold standard measure. Therefore, any quantitative measure of participation in physical activity will be eligible in this review, including both self-report measures (e.g. questionnaires such as the International Physical Activity Questionnaire, activity diaries) and objective measures (e.g. pedometers, accelerometers). Measures of physical activity participation may include, but are not limited to, intensity of physical activity, total minutes of physical activity, total energy expenditure, step count, proportion of people active/inactive, frequency of participation in various types of physical activity, adherence to physical activity, proportion of people meeting physical activity recommendations, proportion of people undertaking active travel and/or attendance at physical activity facilities/services.

### Methods for identification of studies

#### Electronic searches

In accordance with MECIR standards, we will conduct a comprehensive literature search that includes large databases from the multi-disciplinary sciences and biomedical disciplines, specialist bibliographic databases to encompass the field of behavioural economics and databases with economic-specific evidence [36]. We will search the following databases from inception: MEDLINE (Ovid), Embase (Ovid), Cochrane Central Register of Controlled Trials (CENTRAL), the Cumulative Index to Nursing and Allied Health Literature (CINAHL) (EBSCO), Web of Science (Clarivate), Scopus (Elsevier), PsycINFO (Ovid), EconLit (EBSCO), SPORTDiscus (EBSCO) and National Health Service (NHS) Economic Evaluation Database. We will use a combination of free text and indexed terms combined with Boolean operators to search for relevant terms for the population, intervention and outcome components as defined above. We present the search strategy for MEDLINE (Ovid) in the Appendix. We will adapt this strategy for the remaining databases with the help of an academic librarian from the University of Melbourne. The strategy was developed based on terms used within other similar reviews and review protocols [16, 41]. The search will be rerun prior to submission for publication and the results screened for potentially eligible new studies.

#### Searching other resources

We will also search online trial registers including ClinicalTrials.gov (http://www.clinicaltrials.gov/) and the World Health Organization (WHO) International Clinical Trials Registry Platform (http://apps.who.int/trialsearch/default.aspx) for ongoing and recently completed studies. We will use these databases to identify RCTs that meet our study criteria and check this against our list of eligible studies. If an eligible completed study is identified through these trial databases and is unpublished but available in other formats (e.g. a dissertation), we will include it in our systematic review. We plan to screen the reference lists of any relevant systematic reviews and of the eligible studies to identify any additional RCTs that may have otherwise been missed. We will follow the recommendation of Adams et al. [42] to exclude grey literature when an academic field is relatively mature. We acknowledge that the field of behavioural economics and health promotion is newer than other fields; however, a significant amount of academic work has been published in this area. For this reason, we will not systematically search any grey literature or conduct hand searching of journals.

### Data collection and analysis

We will use the Covidence systematic review software (Veritas Health Innovation, Melbourne, Australia; available at www.covidence.org) to facilitate screening, data extraction and risk of bias assessment and EndNote reference manager (EndnoteX6, Clarivate Analytics, Boston; available at http://endnote.com/) to manage search results. Both Covidence and EndNote will be used to manage any duplicate papers.

#### Selection of studies

One researcher (MLNL) will conduct the initial search, followed by a two-step screening process. First, two reviewers (MLNL and MH) will independently screen the titles and abstracts to exclude any publications that do not meet the inclusion criteria. In the event of a disagreement between the reviewers, a third reviewer (RSH) will be consulted until consensus is reached. In the second step, two reviewers (MLNL and MH) will retrieve and independently assess the full text of the potentially eligible studies. Once an article is considered eligible, reviewers will examine author names and trial details to link reports of the same study together and prevent duplicates from being included in the review. Any disagreements over the eligibility of studies will be resolved with the third reviewer (RSH).

### Data extraction and management

Two reviewers (MLNL and MH) will independently extract relevant data from the studies. A third reviewer (RSH) will resolve any discrepancies. We will use a standardised, electronic, pre-piloted form and coding framework to extract relevant study characteristics and outcome data. We will extract the following descriptive data from each study:

- Study characteristics: year of publication, study design, study setting (including country), sample size, funding source(s);
- Participant characteristics: relevant details of health status, age, sex, socio-demographic information;
- Intervention characteristics: name of the intervention, description of comparison (control) intervention, including details on mode, duration and intensity. We plan to use the behaviour change taxonomy to specify behaviour change techniques [43] and the incentive design feature attributes outlined in the Adams et al. framework [34] to describe the unique components of the financial incentive interventions. We will record the extent to which interventions are theory-based using the Michie and Prestwich Theory Coding Scheme [44] and also code for social ecological level categories [45]. The two reviewers (MLNL and MH) will use the Template for Intervention Description and Replication (TIDieR) checklist [33] to assess the quality of intervention reporting (i.e. was sufficient detail given about the intervention to allow for replication?);
- Physical activity outcomes: We will extract all available data on physical activity from the intervention and comparison arm(s) of each study at each measured time point and record the measurement tools used. Outcomes will be grouped (if possible) as short-term (< 6 months after intervention has been completed) and long-term (≥ 6 months after interventions have been completed).

We expect the outcomes of interest will be reported as continuous, categorical or dichotomous data. We will extract frequency counts for categorical and dichotomous data, point estimates for continuous data and include the method of statistical analyses used to calculate these values. Additionally, we will extract corresponding measures of variability (standard deviations, standard errors, *p* value or 95% confidence intervals). To make comparisons at the individual study level, we will calculate the effect estimates including relative measures of risk for dichotomous outcomes (e.g. risk ratios or odds ratios with 95% confidence intervals). For continuous measures, we will analyse data based on the mean, standard deviation (SD) and number of people assessed for the intervention and comparison groups to calculate mean difference (MD) and 95% confidence intervals.

### Assessment of methodological quality of included studies

Two review authors (MLNL and MH) will independently use the Cochrane risk of bias tool [46] to assess the risk of bias. We will consider each domain: (1) random sequence generation (i.e. was a random order used to assign people into intervention and control groups?); (2) allocation concealment (i.e. was the allocated treatment adequately concealed from study participants and staff at the enrolment stage?); (3) blinding of personnel and participants (i.e. were expectations biased due to knowledge of the allocated interventions by personnel and participants during the study?); (4) blinding of outcome assessment (i.e. were the personnel assessing outcomes and analysing data sufficiently blinded to the intervention allocation throughout the intervention?); (5) incomplete outcome data (e.g. how much data are missing from each group? Does the missing data meaningfully affect the results?); (6) selective reporting (e.g. did the authors report results on the pre-specified key outcomes?); and (7) other bias. Citing evidence from the study article, relevant papers or the study author, two reviewers (MLNL and MH) will independently rate each domain as either low risk bias, high risk bias or unclear. In the case of disagreement between the review authors (MLNL and MH), RSH will appraise the study independently and the research team will convene until consensus is reached. The risk of bias for each study will be reported in the ‘summary of findings’ tables.

### Unit of analysis errors

If cluster RCTs are included in a study, we will check for unit-of-analysis errors [47] (i.e. when the unit of analysis is different from the unit of allocation). For example, a study that randomises workplaces to one of several incentive structures (rather than randomising participants) should analyse data at the unit of allocation (i.e. workplace) to account for the similarity between individuals within the same workplace. If authors ignore this intracluster correlation, we will report the effect estimates and note the unit-of-analysis error.

### Missing data

We will attempt to contact the study authors of included studies to request any missing or unclear information including unreported data or data represented only in graphical format. If we are unable to obtain the missing data, we will report an unclear risk of bias for the Cochrane risk of bias domain: incomplete outcome data. We will address the impact of missing data in sensitivity analysis. We will refer to study protocols and baseline publications to identify outcome data that are expected to be present at follow-up. If the data are completely absent (i.e. study findings not published), we will note reporting bias.

### Data synthesis

Where appropriate, we will pool data from studies that are sufficiently similar in population, settings, interventions and clinically relevant outcomes. We will account for the expected heterogeneity among included studies by using a random effects meta-analysis and the inverse weighting method using Review Manager statistical software (RevMan [computer program], version 5, Copenhagen: The Nordic Cochrane Centre, The Cochrane Collaboration, 2014). Where possible, continuous outcomes will be reported on their original scale or as standardised mean differences with 95% confidence intervals. Dichotomous outcomes will be presented as relative risks with 95% confidence intervals.

We will assess the degree of heterogeneity by visually inspecting forest plots and by examining the chi-square test for heterogeneity. Heterogeneity will be quantified using the *I*^2^ statistic, which describes the percentage (0–100%) of total variation across studies [48]. This percentage will be interpreted taking into consideration the size and direction of effects and the strength of the evidence for heterogeneity, based on the *p* value from the chi-square test. Although distinct cut-points do not exist, an *I*^2^ value of greater than 75% is typically considered to be substantially high heterogeneity [49, 50]. Where heterogeneity is present in pooled estimate effects, we will explore the possible reasons for variability by conducting subgroup analysis.

If we are unable to perform a meta-analysis, we will conduct a narrative synthesis of results that considers potential clinical or methodological similarities and differences to explain the heterogeneity between the findings of different studies and examine patterns in the data [51]. We will record all physical activity outcome data. First, these data will be synthesised by intervention ecological category [45]. Since there are a diversity of measurement methods assessing physical activity and no gold standard taxonomy to classify these outcomes [52], we plan to present the outcome data using the domains of the physical activity assessment decision matrix. The tool has both clinical and research relevance and includes outcome types such as ‘meeting physical activity guidelines’ or ‘walking behaviour’ [53]. At minimum, we will synthesise discussion of self-report and objective measures and consider continuous and categorical measures of physical activity separately. We will group together studies that are similar in population (e.g. healthy participants, patients with disease pathology) or have similar intervention features (e.g. positive gain, avoidance of penalty). We also intend to explore if any patterns emerge in comparing theory-driven interventions and atheoretical interventions as defined by the Michie and Prestwich Coding Scheme [44]. We plan to produce forest plots for individual studies (i.e. without a pooled estimate), which is an approach others have used previously in a systematic review including physical activity outcomes [54].

We plan to use the GRADE approach, as described in the Cochrane Handbook [2], to assess the quality of the body of evidence. We will use GRADEpro GDT application (http://gradepro.org) to produce a ‘summary of findings’ table to compare the intervention effect magnitudes on main outcomes. Separate comparisons will be formed by the direction of the financial incentive (positive gain, avoidance of penalty) and comparator (standard intervention).

### Assessment of publication bias

If there are at least 10 studies eligible for review, we will use funnel plots to examine small study effects and highlight the presence of any publication bias. We will perform a statistical test for funnel plot asymmetry and will conduct further statistical investigations to explain any asymmetry.

### Sensitivity analyses

The study authors have prioritised the domains of allocation concealment, blinding of personnel and participants and blinding of outcome assessment as the greatest threats to internal validity for the included studies. We will perform sensitivity analyses to assess the robustness of meta-analytic estimates by conducting one meta-analysis with all eligible studies and another that excludes studies that report an unclear or high risk of bias on any one these domains from the Cochrane risk of bias assessment [46]. This sensitivity analysis has been decided a priori, and we will conduct further sensitivity analyses if necessary.

### Subgroup analyses

We anticipate that our data set will include too few studies to perform subgroup analyses, but if the data allow, we will investigate the intervention effects according to health status (healthy versus disease pathology) and intervention design (financial reward versus avoidance of penalty).

## Discussion

Given the health benefits of physical activity, all adults should engage in 150 min of moderate aerobic exercise or 75 min of vigorous aerobic exercise per week. Nevertheless, few adhere to this global recommendation [2]. Financial incentive interventions use behavioural economic principles to capitalise on decision-making errors that can increase participation in physical activity. To our knowledge, our systematic review will be the first to examine health-promoting financial incentives for physical activity through the lens of a behaviour change system that considers the role of multiple levels of the social ecological model and highlights the quality of intervention reporting. Our systematic review will use an established framework to document financial incentive design features [34], use the TIDieR checklist to evaluate the completeness of trial reporting [33], report specific behaviour change techniques as they apply to the COM-B system for understanding behaviour [10, 43] and evaluate the extent to which studies are theoretically based [44]. The findings will synthesise the research on health-promoting financial incentives and physical activity and contribute to the body of knowledge of behavioural economics and behaviour change theory.

## Appendix

### MEDLINE search strategy

This is the template search strategy that will be adapted as needed to fit the other 12 databases searched for the final review. The exact search for each of the databases will be available on request from the authors at final publication.

Unless otherwise stated, search terms are free text terms; MeSH = Medical subject heading (Medline medical index term); exp = exploded MeSH; tw = text word; adj = adjacent; ti = title; ab = abstract

- [CONCEPT 1: population—all adult]

1. Exp Adult/
2. (Adult or adults or men or women).tw.
3. 1 or 2

- [CONCEPT 2: financial incentives]

4. exp. Reward/
5. Exp Financial Support/
6. Exp Employee Incentive Plans/
7. (award* or reward* or incentive* or payment* or pay or disincentive* or penalty or penalties or voucher*) adj4 (cash or money or monies or monetary or financ* or economic* or fiscal or re-imbursement* or reimbursement* or reinforcement* or tangible* or lump-sum* or lump sum* or material* or individual*).tw.
8. ((deposit or deposits or depositing or commitment or commitments) adj4 (contract or contracts or contracting)).tw.
9. (Pay* adj2 perform*).tw.
10. (P4P or P4P4P).tw.
11. ((conditional or condition or conditions or contingent or contingency) adj2 (cash or money or monies or monetary* or payment or pay* or finance* or management)).tw.
12. ((behavior* or behaviour*) adj1 economic*).tw.
13. ((Competition* or contest or contests or raffle* or lottery or lotteries or prize* or award*) adj4 (cash or money or monies or monetary or financ* or economic* or tangible or lump sum* or lump-sum* or material* or individual* or external or personal or target* or direct* or intervention*)).tw.
14. (Monetary support).ab,ti.
15. (subsidy or subsidies).ab, ti.
16. ((contingent or cash) adj1 payment*).ab, ti.
17. (Deposit contract or deposit contracts or deposit contracting).ab, ti.
18. (subsid*adj4 (physical activity or physical activities or physically active or exercise)).tw.
19. ((economic* or financial or money or monetary or cash) adj4 (assist* or support* or supplement* or transfer*)).tw.
20. (employee incentive plan or employee incentive plans).ab, ti.
21. (employee* N4 (incentive* or rebate* or remuneration* or bonus* or reimburse*)).tw.
22. 4 or 5 or 6 or 7 or 8 or 9 or 10 or 11 or 12 or 13 or 14 or 15 or 16 or 17 or 18 or 19 or 20 or 21

- */And*
- [CONCEPT 3: physical activity]

23. Exp Exercise/
24. Exp Exercise Therapy/
25. Exp Sports/
26. Exp Recreation/
27. Exp Physical fitness/
28. Exp Health Promotion/
29. (increas* or sustain* or encourag* or motivat* or promot* or improv*).ab,ti
30. (23 or 27) and 29
31. (physical activ* or exercis* or aerobic activ* or moderate activ* or vigorous activ* or moderate exercis* or vigorous exercis*).ab,ti.
32. 28 and 31
33. Exercise*.tw.
34. (Physical* adj3 activ*).tw.
35. (Physical* adj3 endurance).tw.
36. (Physical inactivity or physically inactive or sedentary).tw.
37. (physical* adj3 (inactiv* or sedentary)).tw.
38. ((physical activity or physically active or exercise or aerobic) adj3 (bout* or minute* or hour* or day or moderate or vigorous)).tw.
39. (physical activ* or aerobic activ* or moderate activ* or vigorous activ*) adj4 (increas* or sustain* or encourag* or motivat* or promot* or improv*).ab,ti.
40. ((physical exercis* or aerobic exercis* or moderate exercis* or vigorous exercis*) adj4 (increas* or sustain* or encourag* or motivat* or promot* or improv*)).ab,ti.
41. (fitness) adj5 (increas* or sustain* or encourag* or motivat* or promot* or improv*).ab,ti.
42. ((walk* or run* or jog* or yoga or swim* or bicycle$) adj5 (increas* or sustain* or encourag* or motivat* or promot* or improv*)).ab,ti.
43. ((aerobic or strength or resistance) adj3 (train*)).ab,ti.
44. ((Activ* or health*) adj3 (commut* or transport* or travel*)).ab,ti.
45. 24 or 25 or 26 or 30 or 32 or 33 or 34 or 35 or 36 or 37 or 38 or 39 or 40 or 41 or 42 or 43 or 44
46. 3 and 22 and 45

## Additional file

Additional file 1: PRISMA-P 2015 Checklist. (DOCX 32 kb)

## Abbreviations

MD: Mean difference
MECIR: Methodological Expectations of Cochrane Intervention Reviews
PICO: Population, Intervention, Comparison, Outcome
PRISMA-P: Preferred Reporting Items for Systematic Review and Meta-Analysis Protocols
RCT: Randomised controlled trial
SD: Standard deviation
SMD: Standardised mean difference
TIDieR: Template for Intervention Description and Replication
WHO: World Health Organization
